# Mechanistic insights into the antimicrobial activity of a novel essential oil blend against *Bacillus subtilis* using RNA sequencing

**DOI:** 10.3934/microbiol.2026023

**Published:** 2026-08-12

**Authors:** Breyana N. Robinson, Kelechi A. Ike, Amsiray Padilla-Arroyo, Lori N. King, James M. Enikuomehin, John O. Adebayo, Ahmed E. Kholif, Uchenna Y. Anele

**Affiliations:** Department of Animal Sciences, North Carolina Agricultural and Technical State University, USA

**Keywords:** differential gene expression, subinhibitory exposure, oxidative stress, cell envelope stress, metal ion homeostasis

## Abstract

The growing need for alternatives to conventional antibiotics has intensified interest in essential oils as multitarget antimicrobial agents. This study investigated the global transcriptional response of *Bacillus subtilis* to a novel essential oil blend (EOB1) composed of garlic, lemongrass, cumin, lavender, and nutmeg. The minimum inhibitory concentration (MIC) of EOB1 against *B. subtilis* was determined to be 25% (v/v), and a subinhibitory concentration of 12.5% (v/v) was used for transcriptomic analysis. Exposure to EOB1 caused widespread transcriptional changes, with 2178 genes identified as differentially expressed, including 1044 upregulated and 1134 downregulated transcripts. After filtering at a false discovery rate (FDR) of <0.05 and an absolute log2 fold change of >1, 1682 differentially expressed genes remained, comprising 981 upregulated and 701 downregulated genes. This corresponds to approximately 41% of the 4100 annotated protein-coding genes of the *B. subtilis* 168 genome. Principal component analysis showed clear separation between treated and control samples, with the first principal component explaining 97% of the total variance, indicating a strong global transcriptional effect of EOB1 on bacterial gene expression; because library preparation and sequencing were performed in a single batch and replicates within each condition clustered tightly, this separation is attributable to the treatment rather than to technical or batch-related variability. Among the most significantly induced genes were *arsB*, *copA*, *cadA*, *pchE*, *htrB*, *cypX*, *arsC*, and *nfrAB*, suggesting the activation of metal detoxification pathways, oxidative and envelope stress responses, secretion stress pathways, and secondary metabolite-associated responses. In contrast, genes involved in cell wall remodeling and nutrient uptake, including *cwlO* and *gltT*, were strongly downregulated. Gene Ontology enrichment analysis further supported these patterns, with significant overrepresentation of fatty acid β-oxidation, oxidoreductase activity acting on nicotinamide adenine dinucleotide phosphate (NAD(P)H), stress response pathways, amino acid biosynthetic processes, and cytoplasmic components. Collectively, these findings indicate that EOB1 disrupts multiple aspects of the physiology of *B. subtilis* and triggers coordinated metabolic, redox, and envelope-associated stress responses. This study provides mechanistic insight into the antimicrobial activity of EOB1 and supports the potential of essential oil blends as alternative antimicrobial agents.

## 1. Introduction

The rapid expansion of antimicrobial resistance represents a major threat to global health, undermining the efficacy of conventional antibiotics and intensifying the need for alternative antimicrobial strategies. The extensive use of antibiotics across clinical, agricultural, and environmental contexts has accelerated the selection of resistant bacterial strains, thereby complicating infection control and increasing the healthcare burden [1]. Consequently, there is increasing interest in natural bioactive compounds with antimicrobial activity and a reduced propensity for the development of resistance. Among these, essential oils have emerged as promising candidates due to their chemical complexity and multitarget modes of action.

*Bacillus subtilis* is a well-characterized Gram-positive bacterium widely used as a model system and industrial microorganism. It plays a central role in studies of bacterial physiology, stress adaptation, and gene regulation and is extensively applied in enzyme production, fermentation processes, and probiotic formulations. Although generally nonpathogenic, *B. subtilis* exhibits context-dependent adaptive responses that make it a robust system for dissecting antimicrobial effects at the physiological and molecular levels [2]–[4]. Its genetic tractability and well-annotated regulatory networks provide a powerful framework for linking antimicrobial exposure to cellular response mechanisms. Understanding how *B. subtilis* responds to antimicrobial compounds is therefore critical not only for microbiological research but also for improving strategies in food safety, animal production, and industrial microbiology.

Essential oils are complex plant-derived mixtures of volatile secondary metabolites with antimicrobial, antioxidant, and anti-inflammatory activities [5]. Their bioactivity is primarily attributed to constituents such as monoterpenes, phenolics, and organosulfur compounds, which act through disruption of cell membranes, inhibition of enzyme activity, and induction of oxidative stress [6]–[8]. Importantly, their multitarget activity is thought to reduce, though not eliminate, the likelihood of resistance evolution relative to single-target antibiotics, as reduced susceptibility to essential oils and their individual constituents has also been reported [9]. Nonetheless, this multitarget mode of action is generally considered advantageous compared with single-target agents [10]–[13]. Synergistic interactions among essential oils' components further enhance the antimicrobial potency beyond that of individual constituents [14]. Recent evidence shows that a multicomponent essential oil blend comprising garlic, lemongrass, cumin, lavender, and nutmeg (EOB1) exerts strong, concentration-dependent inhibition of both Gram-positive and Gram-negative bacteria, including *Escherichia coli* and *B. subtilis* [5], with a minimum inhibitory concentration (MIC) of 25% (v/v) across all tested species and marked reductions in growth rate, bacterial adaptation, and relative fitness at subinhibitory and higher concentrations. The individual constituents of this blend, garlic and cumin (rich in organosulfur compounds), lemongrass and lavender (rich in monoterpenes), and nutmeg (rich in phenylpropanoids), were selected on the basis of prior evidence of complementary and potentially synergistic antimicrobial mechanisms, which were expected to enhance the overall antimicrobial efficacy relative to any single constituent [14]. Beyond growth inhibition, subinhibitory exposure to EOB1 alters the growth kinetics, including prolonged lag phases and reduced growth rates, suggesting induction of physiological stress states. However, a mechanistic understanding of these effects remains limited, as most studies have focused on inhibitory concentrations and endpoint-based growth metrics rather than the underlying molecular responses.

RNA sequencing now enables high-resolution, genomewide characterization of bacterial transcriptional responses to antimicrobial stress [15]. Transcriptomic profiling facilitates the identification of differentially expressed genes and perturbed pathways, including changes in metabolic, membrane-associated, and transport functions [16]. Studies of bacterial exposure to essential oils have further identified transcriptional changes in genes associated with motility, invasion, and biofilm formation [17]. This systems-level resolution is essential for linking antimicrobial exposure to coordinated regulatory and metabolic changes. Recent research has continued to examine the effects of individual essential oils, including *Litsea cubeba* essential oil, on bacterial regulatory and biofilm-associated pathways [18]. However, the global regulatory impact of defined multicomponent essential oil blends remains insufficiently resolved, particularly under subinhibitory exposure conditions that are relevant to environmental and applied settings. Therefore, the objective of this study was to investigate the global transcriptional response of *B. subtilis* to a defined novel essential oil blend (EOB1), composed of garlic, lemongrass, cumin, lavender, and nutmeg in the ratio 4:2:2:1:1 using RNA sequencing, with the aim of elucidating its mechanistic basis of antimicrobial action. Garlic, lemongrass, cumin, lavender, and nutmeg oils were selected primarily because each has independently demonstrated antimicrobial activity against a range of Gram-positive and Gram-negative bacteria. Differential gene expression and functional enrichment analysis were integrated to characterize the affected pathways and regulatory networks. This study provides a systems-level framework for understanding essential oil-mediated bacterial inhibition and supports their potential development as alternative antimicrobial agents in the context of rising antibiotic resistance.

## 2. Materials and methods

The procedures followed in this study were adapted from and aligned with previously established protocols in the Biology and Animal Sciences Departments [5]. Only modifications relevant to the present experimental objectives are described below; essential parameters governing preparation of the essential oil blend, determination of the MIC, and RNA sequencing are reported directly in the present manuscript to allow independent reproduction without consulting the source publication.

### 2.1. Experimental site

This study was conducted at the Department of Animal Sciences at North Carolina Agricultural and Technical State University, Greensboro, NC, USA.

### 2.2. Preparation of the essential oil stock

The essential oil blend (EOB1) used in this study has been previously characterized [5] using an Agilent 7890 gas chromatography system coupled with a 5977B mass selective detector and a 7693 autosampler (Agilent Technologies, Santa Clara, CA, USA). Briefly, a 2-µL sample was injected at a split ratio of 1:50. The oven temperature program began at 50 °C with a 1-min hold, followed by an increase of 8 °C/min to 220 °C, where it was held for 10 min, yielding a total run time of 32.25 min. Mass spectral data were acquired in scan mode over a range of 50–500 amu with an electron ionization energy of 70 eV. Given the hydrophobic nature of essential oils, a broth microdilution approach was used, in accordance with Clinical and Laboratory Standards Institute guidelines [19], with minor modifications. To obtain the bioactive, water-soluble fraction of the EOB1, equal volumes of the oil and sterile distilled water were combined and mixed thoroughly using an orbital shaker overnight. Following phase separation, the aqueous fraction was carefully collected and used as the experimental working solution. All preparations were performed in independent batches to ensure consistency across replicates. The chemical composition of this aqueous fraction was not independently recharacterized after phase separation, and its constituent profile may therefore differ from that of the original oil blend reported previously [5]; this constraint is acknowledged in the study's limitations.

### 2.3. Bacteria

Ethical approval for this study was granted by the Compliance Committee of North Carolina Agricultural and Technical State University (Approval No. IBC 24-19). *B. subtilis* subsp. *subtilis* str. 168, a Gram-positive bacterium, was evaluated for its susceptibility to the essential oil blend. This strain was chosen because it is a well-established model organism commonly used in antimicrobial and molecular biology studies. The isolate was obtained from the Department of Animal Sciences culture collection at North Carolina Agricultural and Technical State University, Greensboro, NC, USA. Stock cultures were preserved in lysogeny broth (LB) containing glycerol and stored at −80 °C.

### 2.4. Minimum inhibitory concentration

Archived bacterial glycerol stocks were first transferred into an LB medium (Sigma-Aldrich, St. Louis, MO, USA) and incubated overnight at 37 °C under constant agitation at 200 rpm. Following incubation, the resulting culture was adjusted to an optical density (OD_600_) of 0.05 to standardize the inoculum. The MIC was determined using a 96-well microplate containing eight rows (A–H) and 12 columns (1–12). Replicate wells for the bacterial culture were arranged on the same plate. A volume of 200 µL of the EOB1 was dispensed into Column 12, whereas 100 µL of sterile water was added to Columns 1 through 11. Using a multichannel pipette, 100 µL from Column 12 was serially transferred and mixed successively through to Column 3. After the last dilution step, the final 100 µL was discarded, thereby producing a two-fold serial dilution series (performed in independent biological replicates, each measured in technical triplicate wells).

The resulting final EOB1 concentrations in the wells were 50%, 25%, 12.5%, 6.25%, 3.13%, 1.56%, 0.78%, 0.39%, 0.20%, and 0.10% (v/v), corresponding to 500, 250, 125, 64, 32, 16, 8, 4, 2, and 1 µL/mL in the final assay volume. Column 2, which contained no EOB1, served as the positive control. Column 1 contained only LB broth and sterile water without bacterial inoculum and was used as the negative control. Because the material being tested was the aqueous, water-soluble fraction of EOB1 rather than the neat oil, no surfactant or emulsifying agent was required to achieve a homogeneous solution in the assay medium. The microplates were covered with an optically transparent MicroAmp optical adhesive film (Thermo Fisher Scientific®, Waltham, MA, USA) and incubated at 37 °C for 24 h with continuous shaking. To minimize plate position effects, a consistent layout was maintained across replicates in a Varioskan Lux 96-well plate reader (Thermo Fisher Scientific®, Waltham, MA, USA). The MIC was defined as the lowest concentration of EOB1 at which no visible growth was observed, as indicated by the absence of turbidity and no increase in optical density (OD_600_) relative to the positive control. OD_600_ measurements were background-corrected using the negative control wells.

### 2.5. RNA sequencing

To examine how *B. subtilis* responds at the transcriptional level to sub-MIC essential oil treatments, three independent biological replicates (*n* = 3 per group) were prepared for the treatment and the untreated control. Glycerol stocks of *B. subtilis* were first streaked onto LB agar plates and incubated overnight at 37 °C. Individual colonies were then selected to inoculate 3 mL of LB broth and grown at 37 °C with shaking at 200 rpm until the cultures reached the mid-logarithmic phase (OD_600_ ≈ 0.5). At that stage, each culture was treated with the sub-MIC essential oil fraction and incubated for an additional 60 minutes to permit transcriptional changes to occur. This exposure duration was selected because bacterial transcriptomes re-equilibrate within minutes owing to their short mRNA half-lives (~2–5 min) and the rapid activation of the membrane- and envelope-stress regulons targeted by essential oils. Therefore, 60 minutes is sufficient to capture the primary transcriptional response while avoiding secondary effects that accumulate during prolonged exposure [20],[21].

After 60 minutes, each culture was centrifuged at 10,000× *g* for 15 s at 4 °C. The supernatant was discarded, and the bacterial pellets were immediately stored at −80 °C. Frozen samples were shipped on dry ice to SeqCenter (Pittsburgh, PA), where total RNA extraction was carried out, including on-column DNase digestion. Ribosomal RNA was removed using RiboZero, and RNA integrity was verified using a Bioanalyzer, with all samples showing an RNA integrity number (RIN) of at least 8.0. Stranded RNA sequencing (RNA-seq) libraries were prepared using the TruSeq Stranded Total RNA kit and sequenced on an Illumina NovaSeq 6000 platform with 2 × 150 bp paired-end reads, targeting approximately 20 million reads per sample. Library preparation and sequencing were performed in a single batch to minimize technical variability.

After sequencing, adapter sequences were removed from the raw FASTQ files, and low-quality reads were filtered using Trimmomatic. Read quality after trimming was then evaluated with FastQC. The processed reads were mapped to the *B. subtilis* subsp. *subtilis* str. 168 reference genome (NCBI accession NC_000964.3) using HISAT2, and gene-level read counts were obtained with FeatureCounts. Only uniquely mapped reads were retained for downstream analysis. Differential expression analysis was performed using DESeq2 [22], which models read counts with a negative binomial generalized linear model and assesses differential expression using the Wald test. Raw counts were normalized using the DESeq2 median-of-ratios method to correct for differences in library size and composition. Genes were considered differentially expressed if they exhibited an absolute log2 fold change greater than 1 and a Benjamini–Hochberg-adjusted *p*-value (false discovery rate, FDR) below 0.05. The normalized expression values of the differentially expressed genes were used to generate a hierarchically clustered heatmap, and principal component analysis was performed on the variance-stabilized normalized counts of all quantified genes to assess overall transcriptional variation between the treated and control samples.

Sequencing yielded an average of 112 million reads per sample (range: 85–130 million). On average, 98.6% of reads mapped to the *B. subtilis* subsp. *subtilis* str. 168 reference genome (NC_000964.3), the majority of which aligned to protein-coding sequences (mean: 84.1% of total reads), while residual rRNA remained below 0.2%, confirming effective rRNA depletion. Nearly all annotated features were detected (mean: 4203 of 4208), indicating comprehensive transcriptome coverage. Per-sample metrics are provided in Table S1.

## 3. Results

### 3.1. MIC of EOB1 against *B. subtilis*

Over the course of the 24-hour incubation, the essential oil treatment yielded a MIC of 25% (v/v) against *B. subtilis* under study. Based on this result, a subinhibitory concentration (sub-MIC) of 12.5% (v/v), corresponding to 0.5× MIC, was chosen for subsequent experiments to capture transcriptional responses without complete growth inhibition.

### 3.2. Differentially expressed genes

Following exposure to EOB1, *B. subtilis* exhibited widespread and statistically significant transcriptional changes, with a total of 2178 genes differentially expressed, including 1044 upregulated and 1134 downregulated transcripts (Table 1). After applying the significance thresholds of an FDR of < 0.05 and an absolute |log2 fold change| of >1, 1682 genes remained, comprising 981 upregulated and 701 downregulated transcripts. To address the large number of differentially expressed genes, the complete list of all quantified genes and of the significantly differentially expressed genes, including their gene identifiers, expression counts (baseMean), log2 fold changes, and Benjamini–Hochberg-adjusted *p*-values, is provided in Tables S2 and S3, respectively.

**Table 1.** Summary of differentially expressed genes in *B. subtilis* following EOB1 exposure.

Category	Differentially expressed genes	
	Upregulated	Downregulated
All genes	1044	1134
Filtered genes	981	701

RNA-seq analysis revealed substantial upregulation of multiple genes in the EOB1-treated samples compared with the control group. Table 2 shows the top 20 upregulated genes. Among the most strongly induced were *yrkH*, *ohrA*, *yrkI*, and *yrkF*, each showing log2 fold changes near or above 10 along with highly significant adjusted *p*-values. Other notably upregulated transcripts included *arsB*, *copA*, *cypX*, and *pchC*, consistent with a broad transcriptional response in the treated group. The analysis further demonstrated pronounced downregulation of several genes in the EOB1-treated samples compared with the control group (Table 3). Among the most strongly repressed transcripts were *fldN*, *yhcJ*, *ykuO*, and *ylmA*, with log2 fold changes ranging from approximately −6.5 to −7.4 and significant adjusted *p*-values. Other downregulated genes included *walM*, *lytE*, *cwlO*, and *gltT*.

**Table 2.** Top 20 upregulated genes in *B. subtilis* following EOB1 exposure, ranked by log2 fold change.

Gene	Gene/protein annotation	Base mean	Log2 fold change	lfcSE	*P*_adj_-value
*yrkH*	Uncharacterized protein with rhodanese/metallo-β-lactamase-like domains	2875.848	10.609	0.500	5.59E-97
*ohrA*	Peroxiredoxin	90.977	10.289	1.236	5.87E-16
*yrkI*	Uncharacterized protein YrkI	84.080	10.176	1.224	6.36E-16
*yrkF*	Rhodanese family sulfur transferase	246.484	9.871	1.070	2.53E-19
*yrkE*	Uncharacterized protein YrkE	586.688	9.800	0.735	7.05E-39
*yrkJ*	Putative transcriptional regulator YrkJ	116.821	9.687	1.232	2.17E-14
*yhjG*	Uncharacterized membrane protein YhjG	1014.476	9.591	0.589	1.97E-57
*arsB*	Arsenite exporter	3162.658	8.140	0.352	1.24E-114
*ywnB*	Unknown protein YwnB	982.744	8.006	0.404	1.25E-84
*yqcK*	Arsenic resistance-associated protein YqcK	267.932	7.988	0.617	9.84E-37
*hisH*	Imidazole glycerol phosphate synthase glutaminase subunit	100.206	7.985	0.974	1.54E-15
*hisF*	Imidazole glycerol phosphate synthase cyclase subunit	209.349	7.823	0.685	6.64E-29
*hisA*	ProFAR isomerase (HisA)	88.300	7.801	0.952	1.65E-15
*pchC*	Pulcherriminic acid biosynthesis protein PchC	389.570	7.718	0.547	2.58E-43
*hisD*	Histidinol dehydrogenase	137.439	7.703	0.835	2.53E-19
*cypX*	Cytochrome P450, cyclo-L-leucyl-L-leucyl dipeptide oxidase	1062.686	7.687	0.445	1.88E-64
*hisB*	Imidazoleglycerol-phosphate dehydratase/histidinol-phosphatase	78.011	7.622	0.978	3.59E-14
*arsC*	Arsenate reductase	540.059	7.606	0.456	3.38E-60
*copA*	Copper-transporting ATPase	2157.749	7.601	0.352	3.86E-100
*pchR*	Transcriptional regulator PchR	150.227	7.566	0.705	1.09E-25

Abbreviations: baseMean: Mean of normalized counts across all samples; Log2FoldChange: Log2 fold change in expression between EOB1-treated and control groups; lfcSE: Standard error of the log2 fold change estimate; Padj-value: Benjamini–Hochberg-adjusted p-value (false discovery rate).

**Table 3.** Top 20 downregulated genes in *B. subtilis* following EOB1 exposure, ranked by log2 fold change.

Gene	Gene/protein annotation	Base mean	Log2 fold Change	lfcSE	*P*_adj_-value
*fldN*	Flagellar protein FldN	34.569	−7.382	1.231	6.50E-09
*yhcJ*	NlpA-family lipoprotein/ABC transporter-associated protein	94.997	−7.367	0.890	8.20E-16
*ykuO*	Unknown protein	218.846	−6.580	0.528	3.75E-34
*ylmA*	Unknown ABC-transporter-like adenosine triphosphate (ATP)-binding protein	19.314	−6.537	1.245	3.90E-07
*desE*	Putative membrane protein/uncharacterized protein DesE	181.312	−5.956	0.480	6.97E-34
*walM*	WalRK system modulator WalM	22.466	−5.866	1.096	2.32E-07
*fldP*	Flagellar protein FldP	32.905	−5.826	0.910	5.53E-10
*tswB*	Uncharacterized protein TswB	10.486	−5.640	1.268	1.77E-05
*yobQ*	Uncharacterized protein YobQ	10.432	−5.636	1.264	1.68E-05
*tswD*	Uncharacterized protein TswD	5.003	−5.561	1.317	4.71E-05
*lytE*	Cell wall hydrolase LytE	197.456	−5.530	0.430	2.23E-36
*yqaR*	Uncharacterized protein YqaR	4.869	−5.522	1.320	5.58E-05
*cwlO*	Cell wall hydrolase CwlO	1114.749	−5.379	0.306	8.02E-67
*yoaD*	Uncharacterized protein YoaD	4.323	−5.352	1.339	1.19E-04
*gltT*	Major Na^+^-coupled glutamate/aspartate symport protein	1009.044	−5.293	0.339	4.70E-53
*thiU*	Thiamine ABC transporter substrate-binding protein	61.060	−5.283	0.604	1.78E-17
*yoyG*	Uncharacterized protein YoyG	3.959	−5.222	1.345	1.88E-04
*dutA*	dUTP diphosphatase	3.806	−5.168	1.347	2.27E-04
*tboILV*	TboILV transporter-associated protein	54.893	−5.143	0.637	4.16E-15

Abbreviations: baseMean: Mean of normalized counts across all samples; Log2FoldChange: Log2 fold change in expression between EOB1-treated and control groups; lfcSE: Standard error of the log2 fold change estimate; Padj-value: Benjamini–Hochberg-adjusted p-value (false discovery rate).

When ranked by adjusted *p*-value, the most statistically significant differentially expressed genes in the EOB1-treated samples were *arsB*, *copA*, *yrkH*, *ywnB*, and *cadA*, all with exceptionally low adjusted *p*-values ranging from 1.24 × 10^−114^ to 6.55 × 10^−83^ (Table 4). Other highly significant transcripts included *pchE*, *azoJ*, *htrB*, *cypX*, *arsC*, and *nfrAB*, along with the downregulated genes *cwlO* and *gltT*, indicating strong statistical support across both induced and repressed gene sets.

**Table 4.** Top 20 differentially expressed genes in *B. subtilis* following EOB1 exposure, ranked by FDR.

Gene	Gene/protein annotation	Base mean	Log2 fold change	lfcSE	*P*_adj_-value
*arsB*	Arsenite exporter	3162.658	8.140	0.352	1.24E-114
*copA*	Copper-transporting ATPase	2157.749	7.601	0.352	3.86E-100
*yrkH*	Uncharacterized protein with rhodanese/metallo-β-lactamase-like domains	2875.848	10.609	0.500	5.59E-97
*ywnB*	Unknown protein YwnB	982.744	8.006	0.404	1.25E-84
*cadA*	Cadmium-exporting ATPase	14287.183	5.414	0.276	6.55E-83
*pchE*	Pulcherriminic acid synthetase	1126.624	7.297	0.414	7.57E-67
*cwlO*	Cell wall hydrolase CwlO	1114.749	−5.379	0.306	8.02E-67
*azoJ*	Azoreductase	545.015	5.905	0.339	2.44E-65
*htrB*	Putative HtrA-family serine protease/chaperone HtrB	1933.814	4.991	0.287	3.87E-65
*cypX*	Cytochrome P450, cyclo-L-leucyl-L-leucyl dipeptide oxidase	1062.686	7.687	0.445	1.88E-64
*arsC*	Arsenate reductase	540.059	7.606	0.456	3.38E-60
*nfrAB*	Nitro/flavin reductase NfrAB	1404.801	6.893	0.418	5.64E-59
*yhjG*	Uncharacterized membrane protein YhjG	1014.476	9.591	0.589	1.97E-57
*htrA*	Serine protease/chaperone HtrA	4894.917	4.712	0.291	6.71E-57
*uvrA*	Excinuclease ABC subunit A	7554.011	4.630	0.292	1.62E-54
*ywnA*	Uncharacterized protein YwnA	1169.812	5.325	0.336	1.62E-54
*treA*	Trehalose-6-phosphate hydrolase	679.751	5.332	0.338	6.69E-54
*sufL*	Fe-S cluster assembly protein SufL	1952.843	4.933	0.314	1.60E-53
*yhjH*	Uncharacterized protein YhjH	368.144	7.354	0.469	2.85E-53
*gltT*	Major Na^+^-coupled glutamate/aspartate symport protein	1009.044	−5.293	0.339	4.70E-53

Abbreviations: baseMean: Mean of normalized counts across all samples; Log2FoldChange: Log2 fold change in expression between EOB1-treated and control groups; lfcSE: Standard error of the log2 fold change estimate; Padj-value: Benjamini–Hochberg-adjusted p-value (false discovery rate).

### 3.3. Dispersion plot

The dispersion plot (Figure 1) displays the relationship between the mean of normalized counts and gene-wise dispersion estimates. Each black dot represents the initial dispersion estimate for an individual gene, while the red line indicates the fitted dispersion trend. The blue points show the final dispersion values used for differential expression testing, incorporating both gene-wise estimates and the fitted trend. As expected, genes with higher expression levels exhibited lower dispersion, reflecting more stable expression across replicates.

**Figure 1.**
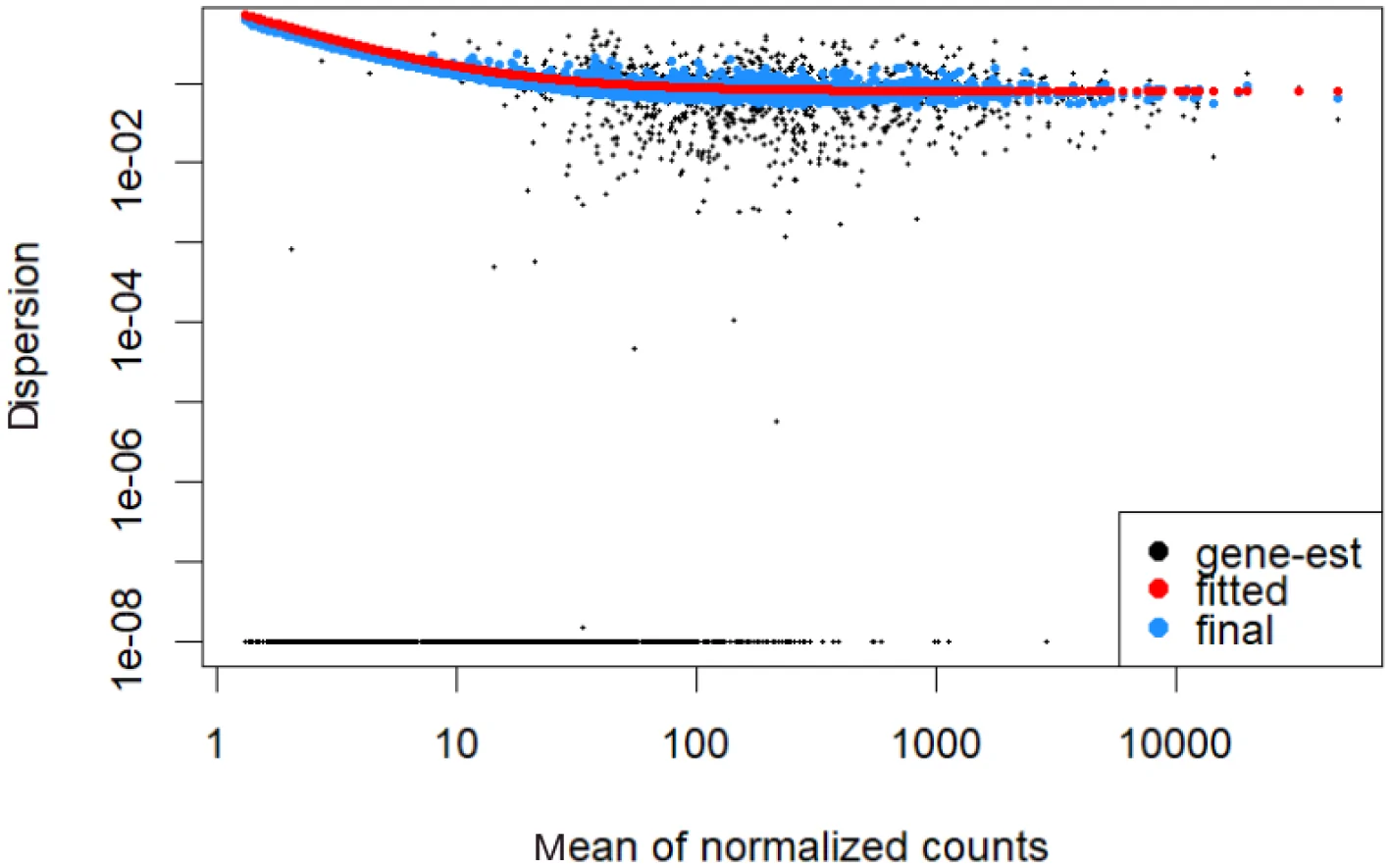
Dispersion estimates for RNA-seq data from *B. subtilis* following EOB1 exposure.

### 3.4. Principal component analysis

Principal component analysis was performed to visualize the overall variance in gene expression profiles between the control and EOB1-treated samples. As shown in Figure 2, the first principal component (PC1) accounted for 97% of the total variance, clearly separating the two treatment groups along this axis.

**Figure 2.**
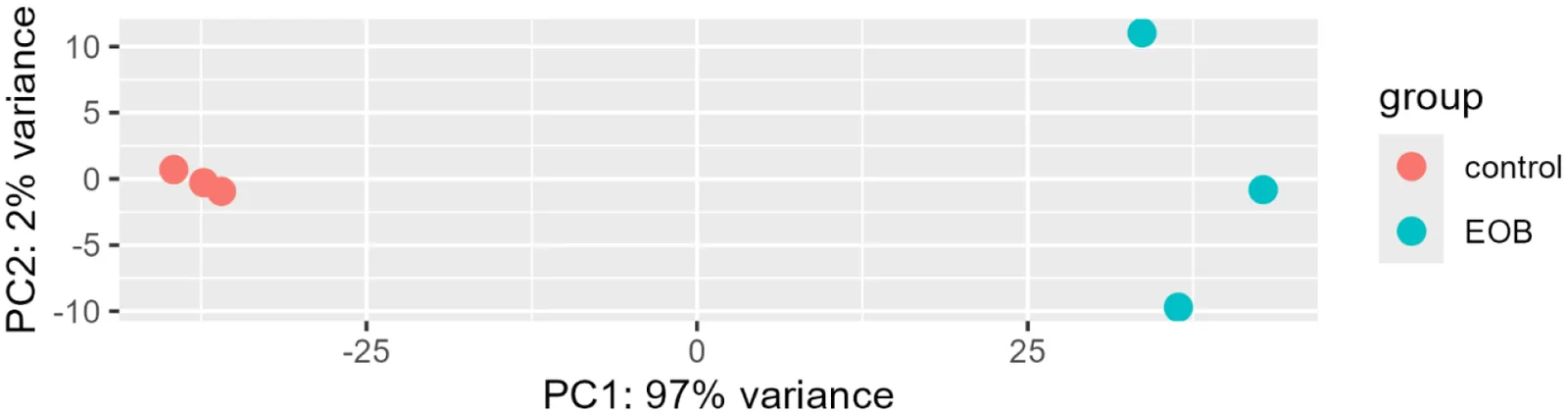
Principal component analysis of RNA-seq samples from *B. subtilis* following EOB1 exposure.

### 3.5. Differential gene expression

To visualize the global changes in gene expression following EOB1 treatment, a volcano plot was generated (Figure 3). Genes with significant upregulation are shown in red, while significantly downregulated genes appear in blue. Transcripts that did not meet the significance threshold are displayed in gray. The plot reveals a clear separation between upregulated and downregulated genes, highlighting the strong transcriptional response triggered by EOB1. Among the most significantly upregulated genes were *arsB*, *copA*, *cadA*, *pchE*, *azoJ*, *htrB*, *ywnA*, *nfrAB*, *cypX*, *arsC*, *yhjG*, and *yhjH*, many of which are associated with stress responses, detoxification, and metal ion transport. Conversely, notable downregulated genes included *cwlO*, *gltT*, *yrhK*, and *ymaA*.

**Figure 3.**
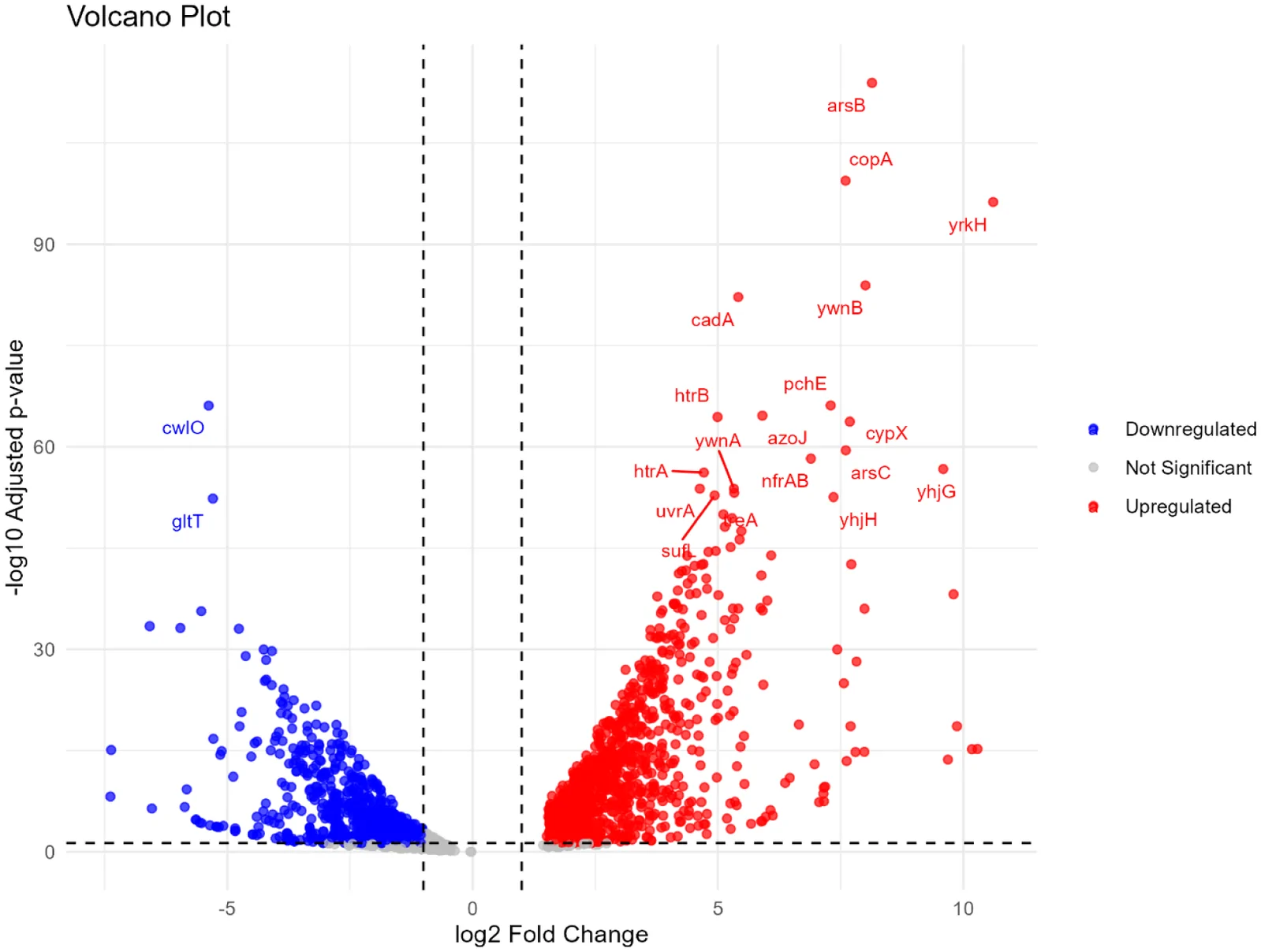
Volcano plot showing differential gene expression in *B. subtilis* following EOB1 exposure. Differentially expressed genes were those with an absolute log2 fold change greater than 1 and a Benjamini–Hochberg-adjusted *p*-value (FDR) below 0.05.

### 3.6. Gene ontology

Gene Ontology (GO) enrichment analysis identified a strong overrepresentation of metabolic and stress-related functional categories among the differentially expressed genes (Figure 4). The most highly enriched terms were fatty acid β-oxidation and the YsiA-like protein, C-terminal region, with a fold enrichment approaching 3.0. Oxidoreductase activity acting on nicotinamide adenine dinucleotide phosphate (NAD(P)H) also showed substantial enrichment, with a fold enrichment greater than 2.5. Notably, the term stress response was significantly enriched, exhibiting both a high fold enrichment and one of the strongest statistical signals based on FDR adjustment. The number of genes contributing to each category is indicated by dot size in Figure 4; enrichment was assessed across the full set of differentially expressed genes (981 upregulated and 701 downregulated), and category sizes ranged from smaller, highly specific terms to the broad metabolic categories, which encompassed several hundred genes each.

**Figure 4.**
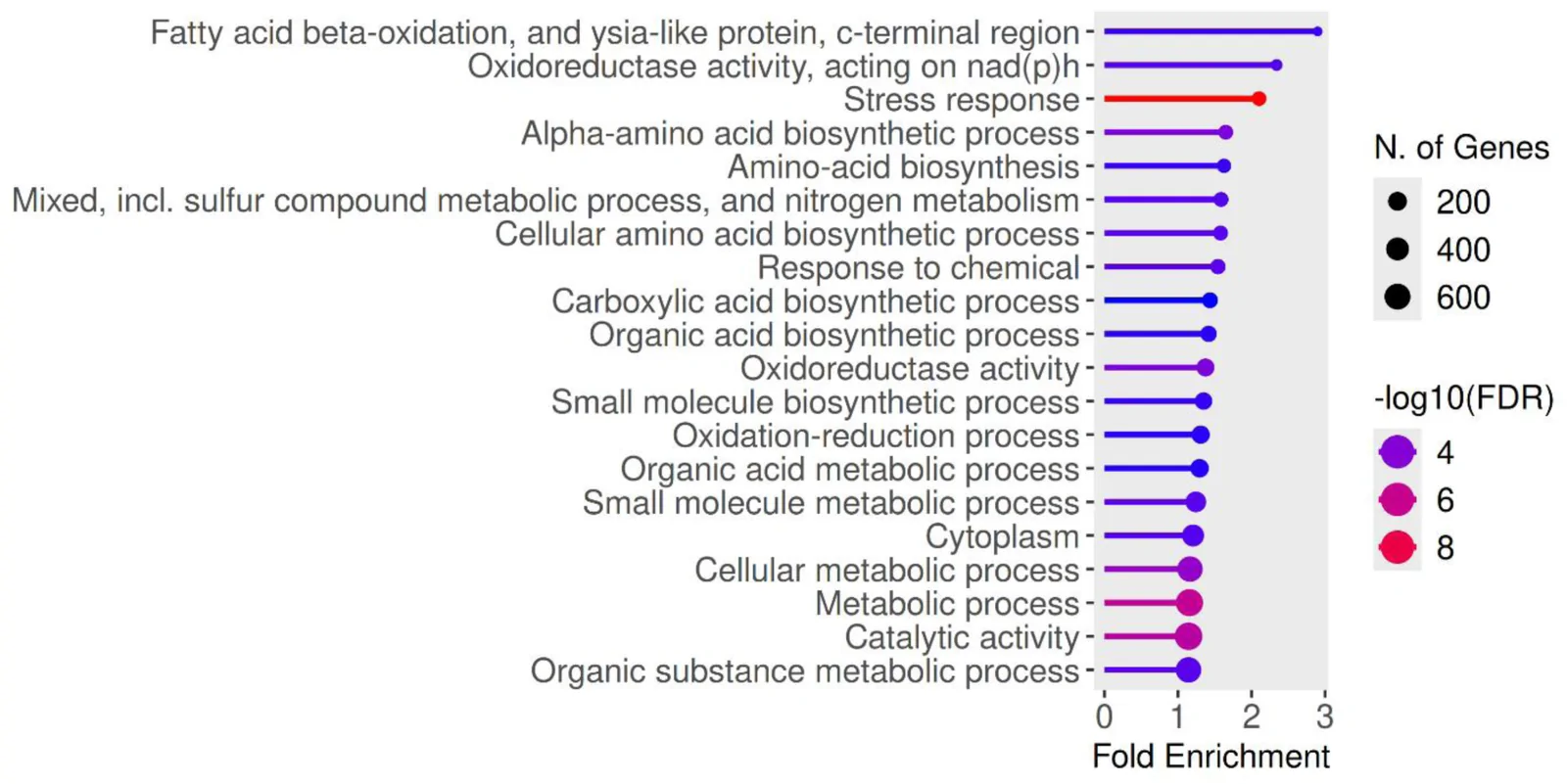
GO enrichment analysis of differentially expressed genes in *B. subtilis* following EOB1 exposure. The *x*-axis represents fold enrichment; dot size indicates the number of associated genes, and dot color represents statistical significance as −log10(FDR) with FDR set at 0.05.

Several biosynthetic pathways were prominently represented. Terms associated with α-amino acid biosynthetic process, amino acid biosynthesis, and cellular amino acid biosynthetic processes all demonstrated fold enrichment values between approximately 1.8 and 2.2. Related categories, including carboxylic acid biosynthetic processes, organic acid biosynthetic processes, and small molecule biosynthetic processes, were similarly enriched. Broader metabolic classifications such as organic acid metabolic processes, small molecule metabolic processes, cellular metabolic processes, metabolic processes, and organic substance metabolic processes were also significantly overrepresented, each with fold enrichment values generally exceeding 1.5. Functional activity categories further supported these findings. Oxidoreductase activity and oxidation–reduction process were enriched, consistent with the prominence of redox-related pathways. Catalytic activity showed strong enrichment and included a large number of genes, indicating widespread enzymatic involvement. The “response to chemical” term was also significantly enriched, suggesting coordinated transcriptional changes in response to environmental or chemical stimuli. In addition, the cytoplasm was enriched as a cellular component, reflecting the predominant localization of many of the identified gene products. With respect to the direction of regulation, the stress- and redox-associated categories, including stress response, oxidoreductase activity acting on NAD(P)H, oxidation–reduction processes, and response to chemicals, were driven predominantly by upregulated genes such as *arsB*, *arsC*, *copA*, *cadA*, *cypX*, *nfrAB*, *htrB*, and *sufU*. This is consistent with the activation of oxidative stress, metal detoxification and envelope stress defense mechanisms. Likewise, fatty acid β-oxidation, the most strongly enriched term, was also dominated by upregulated transcripts. In contrast, cell wall- and nutrient uptake-related genes contributing to the broader metabolic categories, including *cwlO*, *gltT*, *lytE*, and *walM*, were downregulated, whereas the amino acid and organic acid biosynthetic categories comprised a mixture of up- and downregulated genes. A summary of the principal enriched categories, their approximate gene numbers, and their predominant direction of regulation is provided in Table 5.

**Table 5.** Principal enriched GO categories, their domains, approximate number of associated genes (inferred from the dot size in Figure 4), and predominant direction of regulation.

GO term	Domain	Approx. No. of genes	Predominant direction
Fatty acid beta-oxidation/YsiA-like protein, C-terminal region	BP	<200	Upregulated
Oxidoreductase activity, acting on NAD(P)H	MF	<200	Upregulated
Stress response	BP	~200	Upregulated
Response to chemical	BP	~200–400	Upregulated
Oxidation–reduction process	BP	~200–400	Upregulated
Amino acid biosynthetic process (α-amino acid, cellular amino acid)	BP	~200–400	Mixed
Carboxylic/organic/small-molecule biosynthetic process	BP	~200–400	Mixed
Organic acid/small molecule/cellular metabolic process	BP	~400–600	Mixed
Catalytic activity	MF	~600	Mixed
Metabolic process/organic substance metabolic process	BP	~600	Mixed
Cytoplasm	CC	~400–600	Mixed

BP: biological process; MF: molecular function; CC: cellular component. Gene numbers are approximate and were inferred from the dot size in Figure 4; the direction of regulation reflects the predominant contribution of up- or downregulated differentially expressed genes within each category.

## 4. Discussion

The study investigated the global transcriptional response of *B. subtilis* to the essential oil blend EOB1 and demonstrated marked, treatment-associated shifts in gene expression. Differential expression analysis identified more than 2000 significantly altered transcripts, with nearly 1000 upregulated and over 700 downregulated. The large number of differentially expressed genes suggests that EOB1 does not act through a single molecular target but induces coordinated system-wide physiological reprogramming involving stress defense, redox homeostasis, metabolism, membrane function, and regulatory signaling. This is similar to the findings of Yu et al. [23] where *Cinnamomum camphora* essential oil produced 1745, 2311, and 2359 differentially expressed genes in *Escherichia coli* at 1/8, 1/4, and 1/2 MIC, respectively, indicating broad disruption of bacterial regulatory and metabolic processes.

Principal component analysis revealed a clear separation between EOB1-treated and control samples, indicating that the treatment markedly altered the overall transcriptional profile. This separation was driven largely by PC1, which accounted for more than 90% of the total variance, suggesting that EOB1 induced a broad transcriptional shift rather than affecting only a limited set of pathways. The dispersion trend further supported the robustness of the dataset, showing the expected inverse relationship between mean expression and dispersion, in which highly expressed genes displayed lower variability across replicates. Together, these findings demonstrate that the transcriptional response to EOB1 was widespread, reproducible, and indicative of a biologically active complex mixture capable of disrupting multiple aspects of bacterial physiology. Because all samples were processed in a single batch using the same experimental and bioinformatic procedures, and biological replicates clustered closely within groups, the 97% variance explained by PC1 most likely reflects a strong EOB1 treatment effect rather than technical bias.

EOB1 is characterized by a high abundance of both hydrocarbon and oxygenated monoterpenes [5]. Its major constituents, including β-phellandrene (20.3%), β-myrcene (12.1%), terpinen-4-ol (10.5%), geranyl formate (9.4%), p-cymen-7-ol (6.7%), and eucalyptol (6.2%), represent compounds that have been associated with cooperative antimicrobial interactions. Previous studies have shown that phenolic terpenes such as carvacrol become more effective in the presence of monoterpene hydrocarbons like myrcene and p-cymene, which can enhance the penetration of active compounds into the bacterial membrane [14],[24]–[26].

In a similar manner, eucalyptol and terpinen-4-ol inhibit the growth of Gram-positive bacteria at relatively low concentrations, and their combination with other terpenes often produces additive or synergistic effects [27]. Accordingly, the combination of hydrocarbons (β-phellandrene and β-myrcene) with oxygenated monoterpenes (terpinen-4-ol, p-cymen-7-ol, and eucalyptol) in EOB1 likely enhances membrane disruption in *B. subtilis* beyond the effect of any single constituent [28]. This membrane-targeting activity provides a plausible basis for the transcriptomic patterns observed. Disruption of the lipid bilayer may lead to ion leakage and dissipation of the proton motive force, which offers one plausible, though unconfirmed, explanation for the induction of metal efflux systems; because intracellular metal concentrations were not measured directly in this study, this interpretation remains inferential and induction of these genes could alternatively reflect broader, less specific stress-responsive derepression rather than a direct response to metal accumulation [29]. Membrane damage may also interfere with protein secretion and folding, thereby activating the CssRS/HtrA/HtrB secretion–stress pathway, as well as heat shock responses, including the *nfrAB* operon. This interpretation aligns with Hyyryläinen et al. [30], who showed that stress in *B. subtilis* triggered activation of the CssRS regulatory system and strong induction of the *htrA* and *htrB* quality control genes in response to misfolded extracytoplasmic proteins. That observation supports the idea that EOB1-induced envelope disruption may impair protein folding and secretion, thereby provoking both secretion stress and heat shock-associated responses. Similarly, Kachan and Evtushenkov [31] reported that cell wall- and membrane-active compounds, including teicoplanin and polymyxin B, induced the CssRS-regulated htrA response in *B. subtilis*, supporting the view that envelope perturbation can activate secretion stress and protein quality control pathways. At the same time, the combined effects of oxidative and envelope stress may stimulate defense-related and secondary metabolite pathways while suppressing functions that are less critical under stress, such as cell wall remodeling and amino acid transport [32],[33].

Taken together, the chemical composition of EOB1 and the associated transcriptional response suggest that its antimicrobial activity may be driven by synergistic interactions among its constituents, culminating in membrane destabilization and a coordinated, multilayered stress response in the bacterium; because membrane integrity was not assessed directly (for example, by potassium or nucleotide leakage, propidium iodide uptake or membrane potential assays), this membrane-targeting mechanism remains an inference from the gene expression patterns rather than a directly demonstrated effect. A prominent feature of the transcriptomic response to EOB1 exposure was the marked induction of genes associated with metal ion homeostasis and detoxification. In particular, the *ars* operon, which encodes proteins involved in resistance to arsenate and arsenite, showed strong upregulation. Valenzuela-García et al. [34] noted that the arsenic resistance operon within the *B. subtilis* skin element includes *arsR*, *arsI*/*yqcK*, *arsB*, and *arsC*, with ArsR acting as a transcriptional repressor, ArsB as a membrane-associated efflux protein, and ArsC as an arsenate reductase, and that the operon is induced by arsenate, arsenite, and antimonite. Similarly, the *cadA* gene, which encodes a P-type ATPase involved in cadmium export, was upregulated. Previous studies have shown that CadA confers cadmium resistance through an adenosine triphosphate (ATP)-dependent efflux mechanism, supporting its role in active cadmium detoxification. Prabhakaran and Thamarai [35] reported that *cadA* expression in *B. subtilis* confers resistance to cadmium through an ATP-dependent efflux mechanism, suggesting that CadA functions as a proton-exchanging pump.

Upregulation of *copA* and *copZ*, which make up the copper efflux system, was also observed. CopZ acts as a copper chaperone by delivering copper to the CopA ATPase, while CsoR serves as a copper-sensing repressor of the copZA operon, enabling its expression as intracellular copper levels rise [36]. The simultaneous induction of genes involved in arsenic, cadmium, and copper detoxification suggests that exposure to EOB1 may cause intracellular metal accumulation or generate oxidative stress sufficient to stimulate the metal efflux pathways. Because monoterpenes are known to compromise membrane integrity, the resulting increase in permeability may facilitate the entry of metal ions into the cytoplasm, thereby requiring enhanced efflux activity to re-establish cellular homeostasis. It is also possible that essential oils' constituents promote the formation of reactive oxygen species (ROS), which could mobilize intracellular metal pools and activate metal-responsive regulatory networks. Varghese et al. [37] reported that hydrogen peroxide disrupted the activity of the iron-sensing regulator Fur in *E. coli*, leading to downregulation of Fur-controlled genes, which is in line with the possibility that ROS can disturb intracellular metal homeostasis and activate metal-responsive regulatory networks. The study by Méndez et al. [38] indicated that the Scs system links copper homeostasis with redox homeostasis in bacterial pathogens, further supporting the possibility that oxidative stress generated during EOB1 exposure may contribute to the activation of metal-responsive defense systems.

It should be emphasized, however, that intracellular metal concentrations were not measured in the present study; the induction of these operons is therefore consistent with, but does not by itself establish, a change in metal homeostasis, and alternative explanations should be considered. One possibility that does not require altered intracellular metal levels is that reactive, membrane-active constituents of EOB1 directly perturb the cysteine thiol-based sensing residues of the ArsR and CsoR family regulators that control these operons. Because such repressors are derepressed when their sensing thiols are chemically modified, electrophilic or pro-oxidant essential oil components could relieve repression and activate the ars, cad, and cop systems independently of any genuine increase in intracellular arsenic, cadmium, or copper. This form of metal-independent activation would also be consistent with the broader oxidative and electrophilic stress response observed here. Distinguishing between a bona fide change in metal homeostasis and direct chemical activation of these regulators will require a direct quantification of intracellular metal content (for example, by inductively coupled plasma mass spectrometry), which represents an important direction for future work.

Beyond its effects on metal detoxification, EOB1 exposure also provoked a marked stress response associated with protein folding and secretion. One of the prominently induced genes was *htrB*, which encodes an extracytoplasmic protease. In *B. subtilis*, the CssRS two-component system is activated by the accumulation of misfolded secretory proteins and, in turn, stimulates the expression of *htrA* and *htrB*, both of which function in envelope-associated protein quality control [39]. This response helps preserve secretion capacity under stress by removing improperly folded precursor proteins [40]. The induction of these pathways therefore supports the view that EOB1 may impose substantial envelope-associated proteotoxic stress. The *nfrA* and *nfrB* genes were likewise upregulated in the present study. These genes are associated with the heat shock and stress response network, and prior work showed that *nfrA* is strongly induced following thermal upshift, with marked increases in both transcript and protein abundance, supporting its role in cellular adaptation to stress conditions [41]. The study also showed that *nfrA* and the downstream gene are cotranscribed and are not induced by salt or ethanol stress. The induction of *nfrAB* following EOB1 treatment therefore suggests that the essential oil imposes conditions that promote protein damage or oxidative stress similar to those associated with heat shock. Collectively, these findings suggest that EOB1 disrupts protein homeostasis, leading to the accumulation of misfolded proteins and activation of both secretion-related stress pathways and the heat shock response.

Expression of *yrhK*, a component of the SigV-regulated operon, was also increased. The SigV regulon, which includes *sigV*, *rsiV*, *oatA*, *yrhK*, and the *dlt* operon, is induced by lysozymes and contributes to resistance through cell wall modifications, including peptidoglycan O-acetylation and D-alanylation of teichoic acids [42]. The increased expression of *yrhK* therefore suggests that EOB1 may trigger, at least in part, a lysozyme-responsive cell envelope stress pathway associated with protective cell wall remodeling. This is consistent with the broader interpretation that EOB1 may disrupt envelope integrity and activate compensatory defense responses. The increased expression of *pchE* and *cypX*, both of which are involved in secondary metabolite biosynthesis, suggests that exposure to the essential oil triggered a broader adaptive response in *B. subtilis* than growth inhibition alone. In *B. subtilis*, *CypX* has been associated with pulcherriminic acid biosynthesis, a pathway linked to stress adaptation, iron homeostasis, and antimicrobial activity [43]. The upregulation of these genes therefore likely indicates the activation of protective metabolic processes that help *B. subtilis* cope with the stress imposed by essential oil treatment [44]. Cytochrome P450 enzymes are generally involved in oxidative modification of secondary metabolites. The induction of these genes suggests that *B. subtilis* responds to EOB1 by shifting its metabolic activity, possibly enhancing the production of stress-associated or antimicrobial compounds such as pulcherriminic acid. Increased expression of *azoJ* and other uncharacterized oxidoreductases further supports the view that oxidative stress may be an important component of the cellular response.

In this study, several transcripts linked to cell wall remodeling and nutrient uptake were downregulated. Among these was *cwlO*, which encodes a peptidoglycan hydrolase required for cell wall elongation. The ABC transporter-like complex FtsEX is necessary for CwlO's activity and serves as a regulatory system controlling peptidoglycan hydrolases during growth [40]. The reduced expression of *cwlO* may therefore reflect an adaptive response that limits cell wall turnover under conditions of membrane damage, thereby helping preserve structural integrity. Downregulation of cell wall hydrolase activity may represent a protective strategy to reduce further weakening of the envelope during chemically induced stress. Another gene with reduced expression was *gltT*, which encodes a high-affinity transporter for glutamate and aspartate. Zaprasis et al. [45] showed that disruption of *gltT* nearly eliminates glutamate uptake and weakens protection against osmotic stress, indicating its importance in acquiring these amino acids when external concentrations are low. Reduced *gltT* expression may therefore limit osmoprotectant uptake and contribute to growth inhibition. Together, the repression of *cwlO* and *gltT* suggests that EOB1 not only activates stress-defense pathways but also suppresses cellular functions associated with normal growth and nutrient acquisition.

GO enrichment analysis provides a structured framework for interpreting large gene sets by linking them to conserved biological themes across the domains of biological processes, molecular functions, and cellular components [46]. Functional profiling tools are commonly used to test for GO term overrepresentation and apply multiple testing correction, allowing for statistically reliable interpretation [47]. In the present study, the most significant enrichment signals point to metabolic changes closely connected to stress adaptation. The most strongly enriched terms were fatty acid β-oxidation and a YsiA-like protein-related term, both showing fold enrichment values close to 3.0. Oxidoreductase activity acting on NAD(P)H was also markedly enriched, with a fold enrichment above 2.5. Together, these patterns point to a shift toward lipid-associated catabolism and redox rebalancing, responses commonly associated with the disruption of membrane integrity and energy metabolism. Gray et al. [48] showed that membrane depolarization in *B. subtilis* disrupts membrane-associated respiratory activity and promotes ROS production, directly linking membrane perturbation with redox imbalance and energetic stress. In parallel, Schiaffi et al. [49] reported that bacterial fatty acid β-oxidation provides both reducing equivalents and acetyl-CoA for central metabolism, consistent with the idea that, under stress, cells may shift toward lipid-associated catabolism to help restore metabolic and redox homeostasis.

In *B. subtilis*, fatty acid degradation is governed by a dedicated transcriptional regulatory system that is based on FadR, previously described as the YsiA regulon. This regulatory framework may provide a mechanistic explanation for the increased representation of fatty acid β-oxidation-related terms observed in the present dataset. Stress responses were also significantly enriched and showed one of the strongest FDR signals. This finding is consistent with a substantial body of literature indicating that essential oils can disrupt membrane permeability and activate global stress response networks involved in preserving the envelope's integrity and mitigating oxidative stress [6]. Li et al. [7] reported that basil essential oil caused marked damage to the cell wall and cell membrane of *Listeria monocytogenes*, disrupted membrane potential, depleted intracellular ATP, increased ROS accumulation, and altered the expression of oxidative stress-related genes. Yang et al. [50] also showed that lavender essential oil increased ROS production, promoted lipid peroxidation, and enhanced membrane permeability in carbapenemase-producing *Klebsiella pneumoniae*, providing direct evidence that essential oils can simultaneously induce oxidative stress and compromise membrane integrity. In the present study, oxidative stress is inferred from the enrichment of redox-related transcriptional categories rather than from direct biochemical measurement; ROS levels, glutathione or bacillithiol redox status, and lipid peroxidation markers were not quantified, and direct assays of these endpoints would be required to confirm this interpretation.

The enriched biosynthetic terms provide further detail to this pattern. Several amino acid biosynthetic pathways, including the α-amino acid biosynthetic process and the cellular amino acid biosynthetic process, showed fold enrichment values of approximately 1.8 to 2.2, while broader biosynthetic categories involving organic acids, carboxylic acids, and small molecules were also enriched. The simultaneous enrichment of both catabolic and biosynthetic processes is consistent with cells redirecting resources to maintain redox homeostasis, repair cellular damage, and preserve essential metabolite pools under chemical stress [51]. The enrichment of oxidoreductase activity and oxidation-reduction processes also aligns with established redox-buffering mechanisms in Firmicutes, including bacillithiol-dependent systems that protect proteins and support detoxification during oxidative and electrophilic stress [52]. Envelope and secretion stress responses are also biologically plausible in this context, as secretion stress in *B. subtilis* is closely associated with the CssRS-controlled quality-control proteases HtrA and HtrB, which are induced by misfolded or accumulated exported proteins at the membrane–cell wall interface [53],[54].

The induction of pchE and cypX is consistent with activation of the pulcherriminic acid- and pulcherrimin-related pathways. Recent studies have linked these pathways to iron homeostasis, biofilm development, and protection against oxidative stress, suggesting that they may provide an additional layer of stress mitigation during EOB1 exposure [33],[55]–[57]. Across the enriched categories, the relatively high number of genes assigned to each term suggests that EOB1 exposure drives coordinated, system-level remodeling rather than isolated pathway-specific changes. The enrichment of cytoplasmic terms further suggests that many of the responsive gene products are intracellular proteins, including enzymes and regulatory factors, which may help coordinate this adaptive response. Hahne et al. [58] showed that adaptation to salt stress in *B. subtilis* is accompanied by changes in cytoplasmic proteins, including enzymes involved in compatible solute metabolism, central metabolism, and other intracellular stress response functions. These findings suggest that the salt stress response is driven largely by intracellular physiological adjustments rather than being limited to membrane-associated or extracellular changes. Taken together, the GO enrichment profile supports the view that EOB1 exposure does not trigger an isolated stress pathway but instead induces broad metabolic and physiological reorganization consistent with a multitarget antimicrobial mechanism.

***Limitations*:** This study presents some limitations that should be considered when interpreting the findings. The transcriptional response was examined in a single bacterial species (*B. subtilis*), at a single exposure time, and at one subinhibitory concentration, so the results represent only a snapshot of what is likely a dynamic, dose-dependent response and may not generalize to other bacteria. In addition, the findings are based on transcriptomic data alone and the proposed mechanisms were inferred from gene expression patterns and existing literature rather than confirmed through direct biochemical or physiological assays. Complementary approaches such as quantitative real-time polymerase chain reaction, proteomic or metabolomic profiling, and targeted functional assays (e.g., intracellular metal quantification, membrane integrity and membrane potential assays, and ROS measurements) will be needed to confirm the inferred mechanisms. Furthermore, the aqueous fraction of the essential oil blend used for transcriptomic analysis was not independently recharacterized following phase separation; therefore, its chemical composition was assumed, rather than experimentally confirmed, to reflect that of the parent oil blend. Addressing these limitations, including multispecies, time-course, and multiconcentration designs and biochemical validation studies, represents an important direction for future work.

## 5. Conclusions

This study indicated that the essential oil blend EOB1 exerts substantial antimicrobial activity against *B. subtilis* and elicits widespread transcriptional changes even at a subinhibitory concentration. The gene expression profile suggests that EOB1 influences multiple cellular systems rather than acting through a single mode of action. Specifically, at the transcriptional level, EOB1 exposure was associated with altered expression of genes linked to metal homeostasis, redox balance, protein quality control, and cell envelope-associated functions, together with reduced expression of genes involved in cell wall remodeling and nutrient uptake. GO enrichment analysis further reinforced these observations by identifying significant associations with stress responses, oxidoreductase activity, fatty acid β-oxidation, and amino acid biosynthetic processes. Because these proposed modes of action are inferred from transcriptomic evidence and have not been experimentally validated, they should be regarded as hypotheses to be confirmed through targeted functional studies. Collectively, the findings position EOB1 as a promising phytogenic antimicrobial candidate whose activity warrants further investigation at the physiological, biochemical, and applied levels.

## Use of AI tools declaration

The authors declare they have not used artificial intelligence (AI) tools in the creation of this article.


